# Seroepidemiologcal Investigation of Visceral Leishmaniasis in Stray and Owned Dogs In Alborz Province, Central Iran Using Direct Agglutination Test

**Published:** 2013

**Authors:** HR Haddadzade, R Fattahi, M Mohebali, B Akhoundi, E Ebrahimzade

**Affiliations:** 1 Department of Parasitology, Faculty of Veterinary Medicine, University of Tehran, Tehran,Iran; 2 Dept. of Medical Parasitology & Mycology, School of Public Health and Institute of Public Health Research,Tehran University of Medical Sciences, Iran; 3 Center for Research of Endemic Parasites of Iran (CREPI), Tehran University of Medical Sciences, Tehran, Iran

**Keywords:** Canine visceral leishmaniasis, DirectAgglutination Test (DAT), Iran

## Abstract

**Background:**

The aim of present study was to determine the seroprevalence of canine visceral leishmaniasis (CVL) among stray and owned dogs in Kouhsar district of Alborz Province, central Iran.

**Methods:**

The study was performed from March 2011 to July 2011 using Direct Agglutination Test (DAT). Three hundred and thirty seven dogs including 257 stary and 80 owned dogs were selected by random sampling. The agreement between serological data and sex, age, life style of dogs and clinical signs were assessed by Chi-square.

**Results:**

DAT showed that from 337 serum samples collected from owned and stray dogs, 12sera (3.6%) were positive. The seroprevalance was 10% (8/80) among owned dogs and 1.6% (4/257) among stray dogs. A significant difference in seroplevalance was seen between owned and stray dogs (*P* = 0.01). The highest seroprevalence rate (14%) was observed among the ownership dogs of 5 years old and above. Statistical analysis revealed significant relation between seroprelvalence and age (*P*= 0.02). There was no statistically significant relation between male (6.3%) and female (2.2%) seroprevalence (*P*= 0.085).

**Conclusion:**

This survey indicates the importance and necessity of serologic screening of visceral leishmaniasis in human and dogs in Kouhsar district.

## Introduction

Canine visceral leishmaniasis (CVL) is a severe systemic disease of dogs caused by the protozoan parasite *Leishmania infantum* (1). It is transmitted to dogs and human beings via sand fly (2). Every year, approximately 500,000 new cases of visceral leishmaniasis which cause 59,000 human deaths annually, are reported from different parts of the world (3). The disease is endemic in many parts of the world, including Africa, Asia, Europe, the Mediterranean and America. VL is caused by *L. infantum* in the countries of the Mediterranean basin and the Middle East including Iran (4–5). So far in Iran at least four endemic foci of this disease from some areas of Ardabil, East azerbaijan, Fars, Boushehr have been investigated and approved (2–4).

Domestic dogs (*Canis familiaris*) are the main reservoir hosts for human visceral leishmaniasis (3). Approximately half of all *Leishmania* infected dogs lack clinical signs of the disease, but these asymptomatic dogs were shown to be as infective to the vector (sandflies of the genera *Phlebotomus* and *Lutzomyia)* as symptomatic dogs (6). Clinical signs of the disease may develop 3 months to 7 years after infection. Clinical signs usually include lymphadenopathy, dermatitis, alopecia, cutaneous ulcerations, onychogriposis, lameness, anorexia, weight loss, cachexia, ocular lesions, epistaxis, anaemia, diarrhea and renal failure (7). Specific diagnostic techniques are available for canine leishmaniasis including microscopy, culture, serology, polymerase chain reaction (PCR) and xenodiagnosis (4).

Serology is probably the most widely used method for detection of anti-*Leishmania* antibodies in canines, because it is rather easy to perform and provides valuable information in a relatively short time (8).

In the present survey the DAT was used to determine the prevalence of the infection in dogs in studied area, as a simple as well as valid test (3).

## Materials and Methods

### Study area

Kouhsar district is located in Alborz Province of Iran with 543 kilometers in width. The mean elevation of this area is about 1420 m above sea level.

The weather of this district is moderate. This area is near to Taleghan district and Chaloos Road in north, to Hashtgerd new City from west, to Qazvin-Karaj Highway from south and to Karaj City from east. Its population near to be 21790 which 7779 was settled in urban areas and 14011 in rural areas.

### Sampling

The investigation was carried out over a period of 5 months from March 2011 to July 2011 on 337 dogs. Blood samples from 257 stary and 80 owned dogs were taken. From 257 stray dogs’ serum samples, 195 samples were taken from Vafa shelter that located in 2 kilometers of Chendar Village and 62 samples that collected by municipality from different villages of study area. Eighty owned dogs serum samples were taken from 4 village including Chendar, Kordan, Banoo Sahra and Koushk-Zar (each village 20 samples). Before sampling, the information of sex, age and clinical signs were recorded. Blood samples (3-5 ml) were taken from the selected dogs. Serum specimens were separated by centrifugation at 1000g for 5-10 minutes and stored at −20 °C until examined. All the serum samples were tested by DAT in the Leishmaniasis Laboratory in the School of Public Health, Tehran University of Medical Sciences.

### Direct agglutination test (DAT)

DAT for titration of *Leishmania*-specific antibodies followed the general procedures described by Harith (9). The *L. infantum* antigens for this survey were prepared in the Protozoology Unit of the School of Public Health in the Tehran University of Medical Sciences. The principal phases of the method for making DAT antigen were mass production of promastigotes of *L. Infantum* (MCAN/07/IR/Moheb-gh). in RPMI 1640 plus 10% fetal bovine serum, tripsinization of the parasites, staining with coomassie brilliant blue and fixing with formaldehyde 1.2%. Two-fold dilution series were made from 1:80 to 1:20480 in V-shaped micro titer plates (Greiner, Germany) and incubated for 1 hour at 37 °C. Fifty micro litres of reconstituted DAT antigen was subsequently added to each well containing 50 µl of diluted serum. Semi quantitative results obtained with DAT are expressed as an antibody titer, the reciprocal of the highest dilution at which agglutination (large diffuse blue mats) was still visible after 12-18h incubation at room temperature, compared with negative and positive control wells. Specific *Leishmania* antibodies at a titer of 1:320 and above were considered as positive in previous studies. We considered anti *Leishmania* antibodies titers at = 1: 320 as canine *Leishmania* infection in this investigation (1, 9, 10).

### Data analysis

Chi-square was used to compare seroprevalence values relative to gender, age, dogs’ life style and clinical signs. Analyses were conducted using SPSS software version 17 with a probability (*P*) value of <0.05 as statistically significant.

## Results

Sera samples were taken from 337 dogs (257 stary dogs and 80 owned dogs). A total of 12 dogs (3.6%) were seropositive with DAT using a cut-off titer 1:320. Eight dogs from 80 owned dogs (10%) and 4 dogs from 257 stray dogs (1.6%) were seropositive. Significant statistical differences of *Leishmania* prevalence were found between these two groups of dogs in our survey (*P*= 0.01). Seroprevalence values in stray and owned dogs in various age groups are showed in Table 1.

**Table 1:** Seroprevalence of canine visceral leishmaniasis infection in different age-group In stray dogs from kouhsar district, Iran by DAT

Age (yr)	Life style	No. of dogs (%)	DAT test positive. No	Prevalence (%)
	Stray dogs	38 (14.8)	0	0
<1	Owned dogs	9 (11.3)	0	0
	total	47 (13.9)	0	0
	Stray dogs	109 (42.4)	2	1.8
1-3	Owned dogs	24 (30)	3	12.5
	total	133 (39.4)	5	3.7
	Stray dogs	58 (22.6)	2	3.4
3-5	Owned dogs	12 (15)	0	0
	total	70 (20.7)	2	2.8
	Stray dogs	15 (5.8)	0	0
5 <	Owned dogs	35 (43.8)	5	14.3
	total	50 (14.8)	5	10
	Stray dogs	257(76.2)	4	1.6
Total	Owned dogs	80(23.8)	8	10
	total	337(100)	12	3.6

The highest seroprevalence was observed among the owned dogs of above 5 years old. Statistical significance was observed between *Leishmania* infection rates in different age groups of these dogs (*P*=0.02). The seroprevalence values among male and female dogs were 6.3% and 2.2%, respectively (Table 2). No statistically significant differences between canine *Leishmania* infection and gender were observed (*P*= 0.085).

**Table 2:** Seroprevalence of canine visceral leishmaniasis infection in different gender In stray dogs from kouhsar district, Iran by DAT

Life style	gender	Dogs tested,(n)	Relative Distribution (%)	DAT positive	Prevalence (%)
	male	67	62.3	2	2.9
Stray	female	160	21.6	2	1.2
dogs	indeterminate	30	11.7	0	0
	total	257	100	4	1.6
	male	59	73.8	6	10.1
Owned	female	21	26.2	2	9.5
dogs	total	80	100	8	10

Two out of 12 seropositive dogs showed clinical signs including cachexia, skin lesions and weight loss no statically difference was found between *Leishmania* seroprevalence and clinical signs (*P*= 0.148).

## Discussion

Dogs are considered the main domestic reservoir of *L.infantum* for human infection (11). CVL is not only a veterinary problem but it is also a serious public health problem, therefore determination of the prevalence of canine *Leishmania* infection is necessary to define control measures for zoonotic visceral leishmaniasis. Infected dogs, even asymptomatic ones, are sources of infection for Phlebotomine sand flies (3–12).

Since DAT was described as a simple and suitable serodiagnostic test for large-scale screening of CVL in dogs’ population (9), it was used in the present survey. In this study a total of 12 dogs (3.6%) were seropositive. A seroepidemiological study was carried out by Mohebali et al., on 925 serum samples of children under 10 years old and 21 dogs in Kordan region, one child serum and 3 dogs’ sera were seropositive in DAT. In addition in that study, 2090 sand flies were caught in indoor and outdoor of Kordan and Aghasht areas (13).

In Iran, various parts have been shown different seroprevalence rates of *L. infantum* in dogs. The highest seroprevalences rates of CVL have been observed in the Northwest region. The reported seroprevalence rates were 17.4% in 2008 (3), 18.2% in 2005 (5) and 21.6% in 2002 (14). The reported *Leishmania* infection rate in dogs in the Southwest region was 4.4% in 2005 (5).

In the study was carried out by Hosseininejad et al., from 548 dogs serum samples, using DAT in 2011, regarding the sampling regions, seroprevalences of 8.94%, 10% and 16% were found for Tehran, Khoozestan and Chaharmahal-va-Bakhtiari province respectively (15). In Ardestan district in 2012, from 184 owned dogs, twenty dogs (8.1%) were infected, using PCR (16). Seroprevalence rate of visceral leishmaniasis among ownership dogs using IFAT in Sarab district, East Azerbijan was 8.5% (2). In Baft district, Kerman Province in 2010, 7 out of 30 domestic dogs (23%) showed anti-*Leishmania* antibodies at titers ≥1:320 (17). In Khorasan Razavi using IFAT in 2012, the infection rate was 7.6% (18).

The prevalence of the infection in owned and stray dogs in our study were 10% and 1.6% respectively. Statistical differences were found between *Leishmania* infection and dogs’ life style. In Kouhsar district owned dogs are mostly kept in outside, therefore they will be exposed to sand flies bites.

In this survey, the mean age in the owned and stray dogs approximately was 6 and 2.5 years respectively.

In the study by Hosseininejad et al., infection rate was higher in free roaming dogs compared to household dogs (15). This result is inconsistent with our finding. But the high mean age and being more exposure to sand flies bit can be major factors in the high rate of *Leishmania* infection in owned dogs in our study.

We found canine *Leishmnia* infection mostly in older dogs (5 years old and above). In our survey statically difference was observed between dogs older than 5 years and *Leishmania* infection. A seroepidemiological study on 384 serum samples of owned dogs in Meshkin-Shahr district Northwest of Iran in 2007-2008 showed that the seroprevalence of infection was mostly in older dogs (8 years and above) (3). Anti *L.infantum* antibodies increases with age of the dogs (1, 3, 15), these findings are consistent with our result. Seroprevalence in male dogs were more than female but no significant statistical difference was found between canine *Leishmania* infection and gender in our survey. Similar results were found in Portugal, Italy, Greece, and Iran (5, 19, 20, 21). In this survey, only 16% of seropositive dogs showed clinical signs. In other studies in Meshkinshahr district and Fars province 13.6%, 25.4% and 8.1% of seropositive dogs showed a clinical signs respectively (3, 4, 21). This issue has a great importance regarding epidemiology and the transmission of visceral leishmaniasis to human beings, because the dogs without any clinical symptoms similar to the dogs with clinical symptoms have the ability to transmit visceral leishmaniasis to human beings.

## Conclusion

Because of the abundance of sand flies especially *Phlebotomus major* in Kordan district and because of the presence of dogs as a reservoir and source of infection, visceral leishmaniasis must be considered by health authorities in this area (13). This study indicates the importance and necessity of serologic screening of visceral leishmaniasis in human and dogs in Kouhsar district and areas around it.
